# Global Taxonomic Diversity of Anomodonts (Tetrapoda, Therapsida) and the Terrestrial Rock Record Across the Permian-Triassic Boundary

**DOI:** 10.1371/journal.pone.0003733

**Published:** 2008-11-17

**Authors:** Jörg Fröbisch

**Affiliations:** Department of Biology, University of Toronto, Mississauga, Ontario, Canada; Monterey Bay Aquarium Research Institute, United States of America

## Abstract

The end-Permian biotic crisis (∼252.5 Ma) represents the most severe extinction event in Earth's history. This paper investigates diversity patterns in Anomodontia, an extinct group of therapsid synapsids (‘mammal-like reptiles’), through time and in particular across this event. As herbivores and the dominant terrestrial tetrapods of their time, anomodonts play a central role in assessing the impact of the end-Permian extinction on terrestrial ecosystems. Taxonomic diversity analysis reveals that anomodonts experienced three distinct phases of diversification interrupted by the same number of extinctions, i.e. an end-Guadalupian, an end-Permian, and a mid-Triassic extinction. A positive correlation between the number of taxa and the number of formations per time interval shows that anomodont diversity is biased by the Permian-Triassic terrestrial rock record. Normalized diversity curves indicate that anomodont richness continuously declines from the Middle Permian to the Late Triassic, but also reveals all three extinction events. Taxonomic rates (origination and extinction) indicate that the end-Guadalupian and end-Permian extinctions were driven by increased rates of extinction as well as low origination rates. However, this pattern is not evident at the final decline of anomodont diversity during the Middle Triassic. Therefore, it remains unclear whether the Middle Triassic extinction represents a gradual or abrupt event that is unique to anomodonts or more common among terrestrial tetrapods. The end-Permian extinction represents the most distinct event in terms of decline in anomodont richness and turnover rates.

## Introduction

The most severe extinction event in Earth's history at the Permian-Triassic boundary (PTB; ∼252.5 Ma) had a major influence on the diversity of life [e.g. 1]. Between 50–75% of families and 80–95% of species of marine invertebrates and terrestrial vertebrates are estimated to have become extinct [2]. Insect diversity also had its most significant decline at the PTB [3], whereas plant communities showed changes in floral composition rather than loss in total biodiversity [4].

This study focuses on diversity patterns of Anomodontia, an extinct group of terrestrial tetrapods, throughout the Permian and Triassic periods and in particular across the end-Permian extinction event. Anomodonts belong to the diverse clade of Therapsida and constituted the major primary consumers among vertebrates of their time. Therefore, they play a central role for assessing the impact of the end-Permian extinction on terrestrial ecosystems. A well-documented, cosmopolitan fossil record reflects their great taxonomic and morphological diversity, ranging from small burrowing forms to large grazers, which is unparalleled by any other clade of Permian-Triassic terrestrial tetrapods [5]. Extinction rates naturally vary among different clades, leading to the complete extinction of some groups and only a decrease of taxonomic diversity in others. As the dominant element of Upper Permian terrestrial ecosystems, it has often been stated that anomodonts suffered from a substantial decrease in taxonomic diversity at the end of the Permian [6]–[8], before they successfully diversified again in the Triassic.

Diversity studies can be performed on a global or local scale, with both approaches having advantages and disadvantages [9], [10]. For this study, a global perspective is preferred to minimize the influence of biogeographic phenomena, i.e. local extinctions. Furthermore, a broad perspective incorporating a larger time scale enables the differentiation between background extinction and periods of increased extinction.

Methods to estimate global biodiversity patterns can be divided into taxonomic, phylogenetic, and morphological approaches. Phylogenetic diversity measures use a phylogenetic tree topology to infer extended time ranges via ghost lineages, assuming the same age of sister taxa [e.g. 11]. For anomodonts, this approach showed on a short temporal scale that at least four distinct generic lineages survived the end-Permian extinction [12], providing a more complete picture of anomodont survival across the PTB than before. Moreover, within the iconic genus *Lystrosaurus* there are three distinct species, *L. curvatus*, *L. maccaigi*, and *L. hedini*, that are known from below as well as above the Permian-Triassic boundary [13]–[15]. To obtain a complete picture of anomodont survivorship across the PTB it is necessary to also consider their phylogenetic relationships. However, an exhaustive evaluation of the phylogenetic diversity of anomodonts through time was beyond the scope of the previous study and will be investigated elsewhere. A prerequisite for such a comprehensive phylogenetic diversity analysis is the assessment of the taxonomic diversity of this clade, which is the focus of this study.

The present investigation utilizes quantitative methods to assess the taxonomic diversity of anomodonts at the genus and species level. Taxonomic diversity has been extensively studied for many clades. Early studies on the global taxonomic diversity of tetrapods in general and across the PTB were undertaken by Pitrat [16], Bakker [17], Olson [18], [19], Padian and Clemens [20], Benton[e.g. 21], [22], [23], King [7], and Maxwell [8], which in part resulted in substantially divergent conclusions about the impact of the end-Permian extinction on terrestrial ecosystems. In contrast, more recent studies focused exclusively on tetrapod extinction, survivorship, and recovery around the PTB on a local scale [e.g. 24], [25]. A notable exception is a recent study by Sahney and Benton [26], who investigated the taxonomic and ecological recovery of selected terrestrial tetrapod communities after the end-Permian extinction on a global scale. Moreover, Roopnarine et al. [27] recently investigated ecological structures of Permian and Triassic terrestrial tetrapod communities, using simulations of trophic network structures. With respect to anomodonts, the only diversity studies date back more than 15 years [6], [28]. Since then, numerous taxonomic revisions [e.g. 29], [30]–[34] and dozens of descriptions of new taxa [e.g. 35], [36]–[39] have been published. Furthermore, finer stratigraphic resolutions on a local scale as well as correlations between faunas have recently been proposed [40]–[44]. This provides a new framework for an evaluation of diversity patterns of this clade through time. Two prerequisites for such a global taxonomic diversity assessment are a solid faunal correlation and a robust alpha taxonomy. In both areas much work has been done recently for Permian and Triassic anomodonts. The basis for the current study forms an up to date correlation of anomodont-bearing tetrapod faunas with the focus on a well-resolved stratigraphic and taxonomic documentation [13].

The goal of the present study is a global taxonomic diversity assessment (richness, origination and extinction rates) to be performed at genus and species levels. Subsequently, the results are tested for a possible correlation with the Permian-Triassic terrestrial rock record to account for potential sampling biases. Ultimately, this study aims to provide new insights into the taxonomic patterns of extinction, recovery, and diversification of the most diverse and dominant clade of Permian-Triassic terrestrial herbivores and thus the impact of the end-Permian extinction on the continental vertebrate ecosystem.

## Materials and Methods

The diversity assessment of the present study is based on a dataset that has recently been assembled as part of a review of the stratigraphic correlation, composition, and similarity of global anomodont-bearing tetrapod faunas from the Permian and Triassic [13]. Therein, the stratigraphic correlation of global anomodont-bearing tetrapod faunas is mainly based on Rubidge [45] and Lucas [43] for the Permian, while correlation of the Triassic faunas is primarily adopted from Battail [46] and Lucas [42], [47], [48]. Correlation of the well-established South African assemblage zones with the international marine stages and their absolute ages follows Rubidge [45], further supported by Retallack et al. [49]. A definite correlation of the land-vertebrate faunachrons to the international marine stages is at the moment not possible as a result of the scarcity of absolute ages from the terrestrial deposits. However, recent advances in magnetostratigraphy [e.g., 50], [51] and the recovery of precise radiometric ages [e.g., 52], [53], [54] are promising to provide a more robust correlation of tetrapod-based schemes to the SGCS in the future.

**Figure 1:**
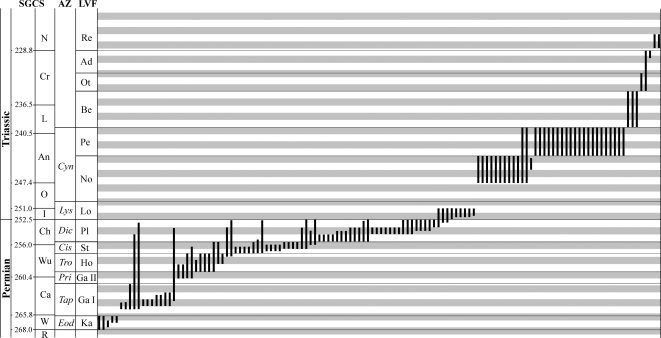
Stratigraphic range chart of Permian-Triassic anomodont species. Grey bars indicate 1 Ma intervals calibrated at the PTB (252.5 Ma), used to calculate diversity at the 1-Ma-scale. Stratigraphic scales include the Standard Global Chronostratigraphic Scale (SGCS), South African assemblage zones (AZ), and Permian-Triassic land-vertebrate faunachrons (LVF). Abbreviations are as follows: Ad, Adamanian; An, Anisian; Be, Berdyankian; Ca, Capitanian; Cr, Carnian; Ch, Changhsingian; *Cis*, *Cistecephalus*; *Cyn*, *Cynognathus*; *Dic*, *Dicynodon*; *Eod*, *Eodicynodon*; Ga, Gamkan; Ho, Hoedemakeran; I, Induan; Ka, Kapteinskraalian; L, Ladinian; Lo, Lootsbergian; *Lys*, *Lystrosaurus*; No, Nonesian; N, Norian; O, Olenekian; Ot, Otischalkian; Pe, Perovkan; Pl, Platbergian; *Pri*, *Pristerognathus*; Re, Revueltian; R, Roadian; St, Steilkransian; *Tap*, *Tapinocephalus*; *Tro*, *Tropidostoma*; W, Wordian; Wu, Wuchiapingian. For an explanation of anomodont taxon ranges see supplementary Text S1.

The current study makes a number of assumptions that the reader needs to be aware of, including the uncertainty regarding the correlation of the LVFs to the SGCS. This concerns the analysis at the stage-scale as well as at the numerical time scales using one and five million-year intervals, respectively (see below). Furthermore, the stratigraphic ranges of the anomodont taxa are not necessarily precisely known, because not all occurrences are tied to measured stratigraphic sections. The listed stratigraphic ranges (Fig. 1) are taken directly from the literature and are discussed in detail elsewhere [13]. In addition, there is a potential taxonomic bias of anomodonts towards Permian taxa, which received considerably more attention recently. Therefore, future taxonomic revisions of Triassic anomodonts might reveal an actually decreased richness when compared to this study.

The present dataset incorporates a total of 128 species in 68 genera from 77 faunal assemblages, spanning a time period of 42 Ma (Fig. 1). The focus of the diversity study is on taxonomic richness, as abundance data is at this point not available for the majority of the taxa. Richness changes through time were investigated on the genus (g) and species (s) level by dividing the Permian and Triassic into four different sets of time bins. The first set of time bins are the international marine stages from the Standard Global Chronostratigraphic Scale (SGCS). The second set of bins is represented by the Permian-Triassic land-vertebrate faunachrons (LVF) established by Lucas and colleagues [42], [43], [48], [55]–[58]. For the purpose of this study, the Permian Gamkan LVF was further subdivided into Gamkan I (*Tapinocephalus* AZ) and Gamkan II (*Pristerognathus* AZ) to reflect the finer stratigraphic resolution of the South African Karoo Basin and correlated strata. The duration of the LVFs varies between 1.5 and 6.5 Ma with a mean duration of 3.19 Ma (3.71 Ma in the Triassic vs. 2.58 Ma in the Permian). To avoid a bias of the diversity patterns by the unsteady duration of the sampling intervals, the third and fourth sets of time bins uses one million year and five million year intervals, respectively. The stratigraphic framework is based on the most recent Geologic Time Scale [59] that was updated based on recently published radiometric ages [e.g. 52], [53] and an updated time scale for the Triassic [60]. The 1-Ma and 5-Ma time scales are calibrated at the Permian-Triassic boundary with an age of 252.5 Ma [adopted from 53], to reflect the faunal turnover across this event most accurately.

Among diversity assessments, richness is usually calculated on the basis of up to five variables [e.g. 61], [62]. Following the terminology of Foote [63], the four main components are taxa that are confined to a single time interval [NFL, ‘singletons’ sensu 64] and three kinds of boundary crossing taxa. The latter comprise taxa that cross the bottom boundary of an interval and have their last occurrence within the interval (N_bL_), taxa that have their first occurrence within an interval and cross its top boundary (N_Ft_), and those that cross the bottom and top boundaries of an interval (N_bt_). The fifth variable is represented by taxa that are known before and after an interval but not within it, also known as Lazarus taxa. For anomodonts this variable is negligible and not further considered. Various combinations of these variables result in different richness counts that respond differently to biases (see below). For example, the total diversity is given as N_TOT_ = N_bL_+N_Ft_+N_bt_+N_FL_.

A large number of potential sampling biases have been proposed to effect diversity assessments, of which some or all have previously been investigated for selected datasets. These include a possible taxonomic bias, time scale bias, collection bias, rock record bias, population bias, research effort bias, and taphonomic bias. With respect to a taxonomic bias, Wagner et al. [65] reported for Paleozoic gastropods, Jurassic bivalves, and Cenozoic bivalves that taxonomic standardization can amplify diversity patterns in some cases, but it does not greatly change inferred richness [see also 66]. This indicates that the effects of taxonomic biases are negligible, if the relative pattern of diversity changes through time is observed.

Foote [63], [67] discussed that greatly varying interval durations within a set of time bins (time scale bias) can misrepresent diversity counts considerably. It is possible to minimize this bias by considering the finest time scale possible or using time bins that are evenly divided. In addition, Foote [63], [67] recommended the exclusive usage of boundary crossing taxa in diversity analyses to circumvent problems associated with interval length. In contrast, Uhen and Pyenson [66] argued against this approach for their cetacean dataset, because almost 50% of the incorporated taxa (genera) therein represent singletons. Depending on the stratigraphic resolution, this concern also applies to anomodonts with for example 82% of the species and 72% of the genera occurring in only one of the 13 LVFs, as opposed to 16% of the species and 12% of the genera that are present in only a single one million year interval. Therefore, to discuss the effect of a possible time scale bias, total anomodont richness (N_TOT_) is investigated in the framework of four divergent sets of time bins, i.e. stages, LVFs, as well as one and five million year intervals (see above). However, with respect to the Middle to Late Triassic the 1-Ma-scale utilizes a higher stratigraphic resolution than is provided by the current correlation scheme. Therefore, it needs to be clarified that the richness curves during the Triassic are artificially high (see discussion below and dashed lines in selected figures).

In addition, at the 1-Ma-scale two further diversity assessments were performed, one excluding singletons (N_TOT_−N_FL_) and one by calculating the estimated mean standing diversity [EMSD, see 63], which reflects the average number of taxa at any given point in time in the interval. In the latter case, singletons are excluded, bottom and top boundary crossing taxa count as one unit, and boundary-crossers with their first or last appearance data but not both within one interval (single-ended taxa) count as a half unit only:
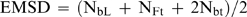



This approach avoids an underestimation of taxonomic rates, which would be the result of a solitary exclusion of singletons. Furthermore, it avoids an artificially high diversity that can be caused by a high turnover rate (origination and extinction) within an interval.

Regarding taphonomic biases, it is well established that different environments and preservational conditions can result in different relationships between live richness and death richness [e.g. 68]. The exclusion of singletons also controls for possible taphonomic biases, such as the Lagerstätten effect [e.g. 69]. However, Fitzgerald and Carlson [70] argued that singletons do not necessarily bias the results of diversity analyses and that singleton taxa potentially reflect rapid evolutionary turnover of taxa. To accommodate either view, separate richness curves are plotted at the 1-Ma-scale including and excluding singleton taxa. A population bias assumes a positive correlation of human population size and fossil findings in a given area. This was investigated for selected cetacean-bearing regions by Uhen and Pyenson [66], who found no discernible relationship between the two variables, rendering a potential population bias insignificant.

In contrast, a research effort bias and most significantly an available rock record bias have previously been reported to have an effect on diversity assessments. For example a positive correlation of rock outcrop area, rock volume, or number of formations with taxonomic richness has been observed for a number of samples [e.g. 71], [72]–[75]. For the purpose of the present study, the possible effect of a rock record bias on the observed anomodont diversity is investigated by testing for a potential correlation between the number of anomodont-bearing formations and anomodont richness in a given time interval. As abundance information is at this point not available for anomodonts, sampling standardization methods (e.g. rarefaction, UW, OW, O2W), which can translate into different diversity curves [see 61], could not be applied to this dataset and will have to await future considerations.

Origination and extinction rates were calculated for the international marine stages and the one million year intervals using Sepkoski's [76] per-taxon rate (r_O_ and r_E_). Thereby, the origination rate (r_O_) is calculated as the number of originating taxa (N_FL_+N_Ft_) divided by the total number of taxa (N_TOT_) within one interval. For extinctions, the per-taxon rate (r_E_) represents the number of taxa becoming extinct (N_FL_+N_bL_) divided by the total number of taxa (N_TOT_) within one interval. At the stage level, origination and extinction rates per interval were further divided by the duration of each stage (Δt). Van Valen metrics for origination and extinction were also calculated but are not illustrated as they show a matching pattern to the per-taxon rates [77], [78]. Estimated per-capita origination and extinction rates [see 63], could not be measured for all the intervals, as some intervals contain N_bt_, N_bL_, and/or N_Ft_ values of zero. In addition, the rates of diversification (r_D_ = r_O_−r_E_) and turnover (r_T_ = r_O_+r_E_) were calculated to establish the overall change in the composition of anomodont faunas throughout the Permian and Triassic.

## Results

### Diversity patterns of anomodonts

Anomodonts experienced three distinct extinctions during the Permian and Triassic, at the end of the Guadalupian, the end of the Permian, and close to the Anisian-Ladinian boundary. This is indicated by analysis of the raw richness data at the stage-, LVF-, 5-Ma- and 1-Ma-scales, including investigations of the total richness at all scales and furthermore total richness without singletons (N_TOT_−N_FL_) and the estimated mean standing diversity (EMSD) at the 1-Ma scale only.

**Figure 2:**
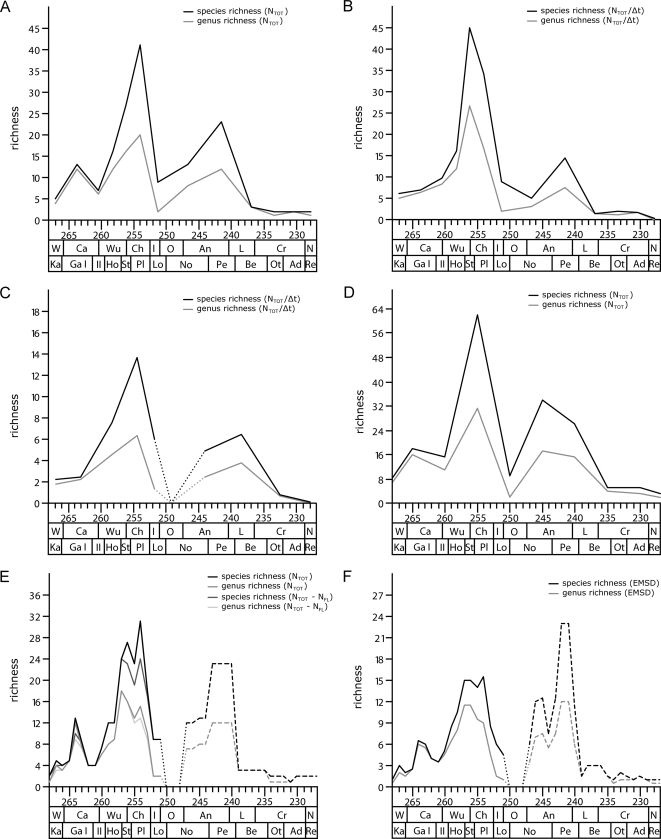
Anomodont diversity. Genus and species diversity curves of a) total richness (N_tot_) at the LVF-scale, b) total richness at the LVF-scale divided by the duration of the LVF (N_tot_/Δt), c) total richness at the stage-scale divided by the duration of the stage (N_TOT_/Δt), d) total richness (N_TOT_) at the 5-Ma-scale, e) total richness (N_tot_) and excluding singletons (N_tot_−N_FL_) at the 1-Ma-scale, and f) estimated mean standing diversity (EMSD) at the 1-Ma-scale. Dotted lines indicate an artificial low in richness as a result of the gap in the fossil record of anomodonts in the late Early Triassic (Olenekian). Dashed lines indicate an artificially increased richness caused by the coarse correlation of Triassic anomodont faunas at the 1-Ma-scale.

Anomodont diversity at the LVF-scale (Fig. 2a) shows that total richness (N_TOT_) increases from initially four genera and five species to twelve genera and thirteen species in the late Guadalupian (Gamkan I LVF). In the early Lopingian (Gamkan II LVF), genus and species diversity is cut in half, but thereafter increases continuously until it reaches its maximum of 20 genera and 41 species in the latest Permian Platbergian LVF (Changhsingian). At the end of the Permian, global anomodont diversity collapses down to only two genera and nine species in the earliest Triassic Lootsbergian LVF (Induan and early Olenekian), suggesting a major impact of the end-Permian extinction on terrestrial tetrapod ecosystems. Recovery of the global anomodont diversity after the PTB lasted several million years, but diversity increased continuously to reach a second peak of twelve genera and 23 species in the Perovkan LVF (late Anisian and early Ladinian). Thereafter, anomodont diversity rapidly collapsed for a third time at the Anisian-Ladinian boundary and remained low, not exceeding three genera and species until the extinction of the clade in the late Triassic (Norian).

The diversity pattern at the LVF-scale changes slightly when corrections for the unequal durations of the LVFs are made (Fig. 2b). First, the diversity peak in the late Guadalupian (Gamkan I) and the subsequent low in the early Lopingian (Gamkan II) disappear and are replaced by a slow and continuous increase in genus and species richness towards the Late Permian. Second, the diversity peak in the Late Permian shifts from the latest Permian Platbergian LVF to the slightly earlier Steilkransian LVF. This results in a decrease in anomodont diversity already in the latest Permian, reaching a minimum in genus richness in the earliest Triassic (Lootsbergian LVF) and a minimum in species diversity in the Nonesian LVF. The diversity peak in the Perovkan LVF and the subsequent final decline of anomodonts mirrors the patterns also seen in the uncorrected curve (Fig. 2a).

Anomodont diversity at the stage-scale (Fig. 2c) was also corrected for the unequal interval length and shows an almost identical pattern seen in the corrected richness at the LVF-scale. Thereby, the richness curve depicts only the end-Permian and mid-Triassic events. However, the overall diversity peak is in the latest Permian (Changhsingian) rather than the early late Permian (Wuchiapingian).

In contrast, the diversity pattern at the 5-Ma-scale (Fig. 2d) depicts all three extinctions, at the end of the Guadalupian (Capitanian), at the end of the Permian, and in the Middle Triasssic. The overall diversity peak is in the latest Permian and the end-Permian extinction represents the most severe event of the three extinctions in anomodont history.

At the 1-Ma-scale, the diversity curve using the total richness (N_TOT_) of anomodonts (Fig. 2e) shows the three peaks and extinctions described for the time-uncorrected richness curve at the LVF-scale and the curve at the 5-Ma-scale. Differences to the previously described diversity curves include a slightly stepped initial diversification to reach the first maximum between 264.5 and 263.5 Ma. The subsequent drop in richness is on the one hand more drawn out, but also more pronounced to only four genera and species between 262.5 and 260.5 Ma at the 1-Ma-scale. Thereafter, the rapid increase in anomodont richness towards the overall maximum in the latest Permian is at first continuous, but shows a slight drop in genus and species richness just before the maximum peak (254.5 to 253.5). The latter is with 15 genera and 31 species overall lower than indicated by the LVF-scale. In addition, the maximum richness at the genus level differs from that at the species level, and is already in the late Permian Steilkransian LVF (257.5 to 256.5), a pattern that was also apparent from the time-corrected richness at the LVF-scale (Fig. 2b). Moreover, the drastic decrease in anomodont diversity at the end of the Permian already starts in the latest Changhsingian (253.5 to 252.5 Ma) before the PTB. The diversity minimum in the earliest Triassic (early Induan) is followed by a gap in the anomodont fossil record lasting approximately three million years (250.5 to 247.5 Ma) for the entire Olenekian. Thereafter, anomodont richness increases continuously to the Triassic diversity peak in the late Anisian and early Ladinian, where it forms a plateau (see below). The final decline of anomodonts shows the same pattern seen at the previous curves.

The diversity curve without singletons at the 1-Ma-scale is also illustrated in Figure 2e and shows the same pattern as the previous one. At this scale, singletons at the genus and species level are only present in the Permian. Their exclusion mainly results in lower peaks at the end of the Guadalupian and the Late Permian Changhsingian. In fact, the overall maximum of anomodont species richness in the latest Permian Platbergian LVF (254.5 to 253.5) is reduced to the same level as in the Steilkransian LVF (257.5 to 256.5), which also corresponds in richness to the Triassic peak.

The estimated mean standing diversity at the 1-Ma-scale as depicted in Figure 2f reflects a further modification of the pattern of anomodont diversity throughout the Permian and Triassic. The overall outline of peaks and lows in anomodont diversity are very consistent with the previous curves. Nonetheless, there are some notable differences that require further discussion. The Permian diversity curve describes the same pattern noted earlier, displaying a slightly stepped increase towards the first distinct peak in the late Middle Permian, a substantial drop in diversity thereafter, and a continuous increase to the peak in the Late Permian Changhsingian. However, the latter peak is even more rounded and the maximal estimated mean standing diversity reflects lower richness values. In contrast, the Triassic diversity curve retains some of the original maximal values, which results in the positioning of the highest peak of overall anomodont richness in the Middle Triassic and not at the end of the Permian. Furthermore, the Triassic curve shows a distinct alteration from previous patterns, in that the peaks are narrower and the continuous increase and decrease towards and after the Triassic maximum is interrupted by periodic diversity drops.

### Anomodont diversity and the rock record

The rock record is known to have a strong influence on the observed diversity in the marine realm [e.g. 71], [72]–[75]. The present study investigates on a global scale if this holds true for the terrestrial rock record of the Permian and Triassic and the diversity therein.

**Figure 3:**
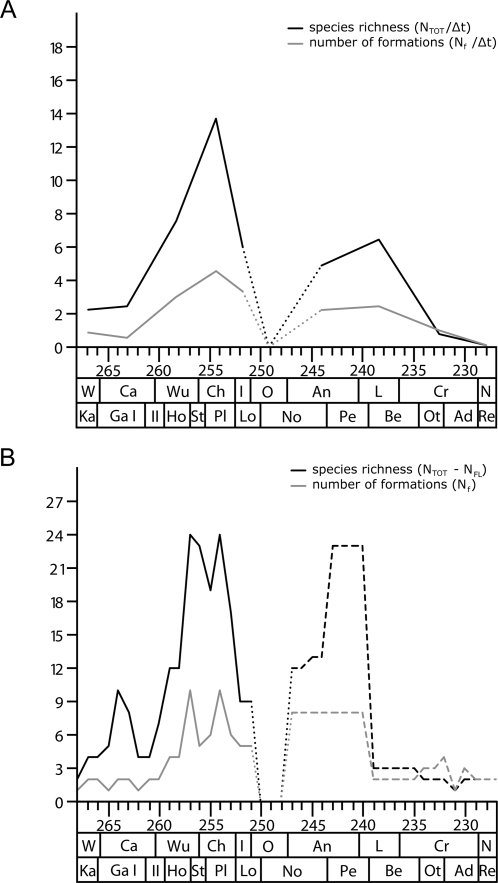
Anomodont species diversity and the rock record. a) Species diversity curve (N_TOT_/Δt) and the number of species informative formations (N_f_/Δt) at the stage-scale, and b) species diversity curve excluding singletons (N_tot_−N_FL_) and the number of species informative formations (N_f_) at the 1-Ma-scale. Dotted lines indicate an artificial low in richness as a result of the gap in the fossil record of anomodonts in the late Early Triassic (Olenekian). Dashed lines indicate an artificially increased richness caused by the coarse correlation of Triassic anomodont faunas at the 1-Ma-scale.

The diversity pattern of anomodonts described above shows three main peaks and subsequent lows in richness. Figure 3 illustrates this pattern throughout the Permian and Triassic, as depicted by the total number of anomodont species at the stage-scale (Fig. 3a) and by the total number of anomodont species without singletons at the 1-Ma-scale (Fig. 3b). When the number of anomodont-bearing tetrapod faunas that are informative on the species level is plotted on the same graphs, these curves reflect a similar but not identical pattern as seen in the associated richness curves (Fig. 3). More specifically, at the 1-Ma-scale, the curve reflecting the number of formations per interval shows the same double peak in the Late Permian, a subsequent low in the Early Triassic, and a Triassic peak in the Middle Triassic followed by the final decline in the Late Triassic. The main differences between the two curves relate to the early part of the plot in the Middle Permian and portions of the Triassic course of the curves. Most importantly, the number of formations does not increase at the end of the Guadalupian, but remains at a low level alternating between one and two formations per sampled interval throughout the Middle and early Late Permian. In addition, the Triassic peak forms a stable plateau throughout the entire Anisian (247.5 to 239.5 Ma), not mirroring the stepped increase of taxonomic richness to a maximum in the late Anisian and early Ladinian.

**Figure 4 pone-0003733-g004:**
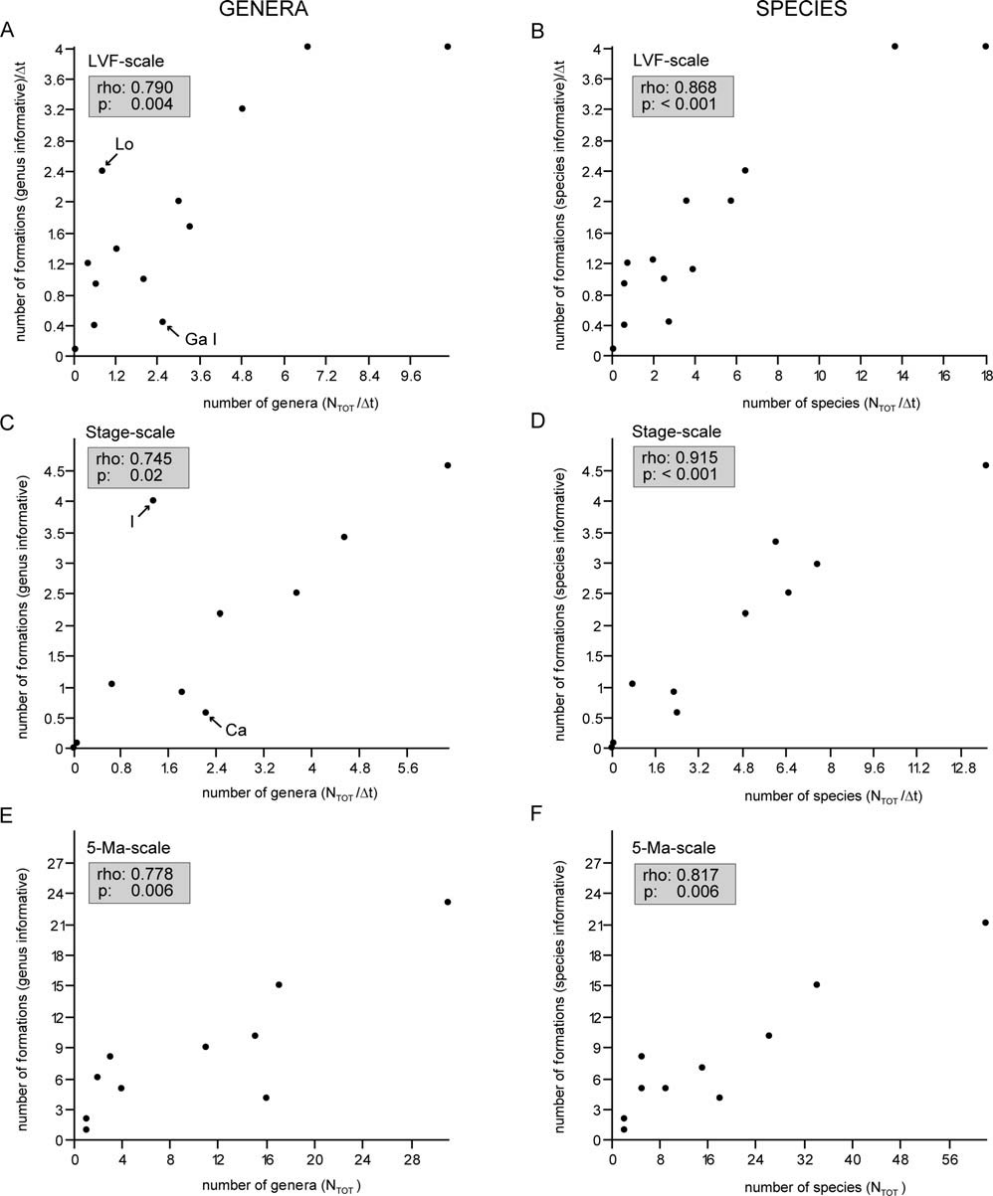
Correlation of anomodont diversity and the rock record at various scales. Graphs plotting the number of genera (a, c, e) and species (b, d, f) against the number of informative formations. a–b) Total richness (N_tot_/Δt) at the LVF-scale, c–d) total richness (N_TOT_/Δt) at the stage-scale, and e–f) total richness (N_TOT_) at the 5-Ma-scale. Spearman's rho and p-values of all variables indicate a statistically significant positive correlation between the number of taxa and the number of formations. Obvious outliers in the genus plots at the LVF-scale and the stage-scale are from the earliest Triassic (Stage: Induan; LVF: Lootsbergian) and from the Middle Permian (Stage: Capitanian; LVF: Gamkan I)(see text for discussion).

**Figure 5:**
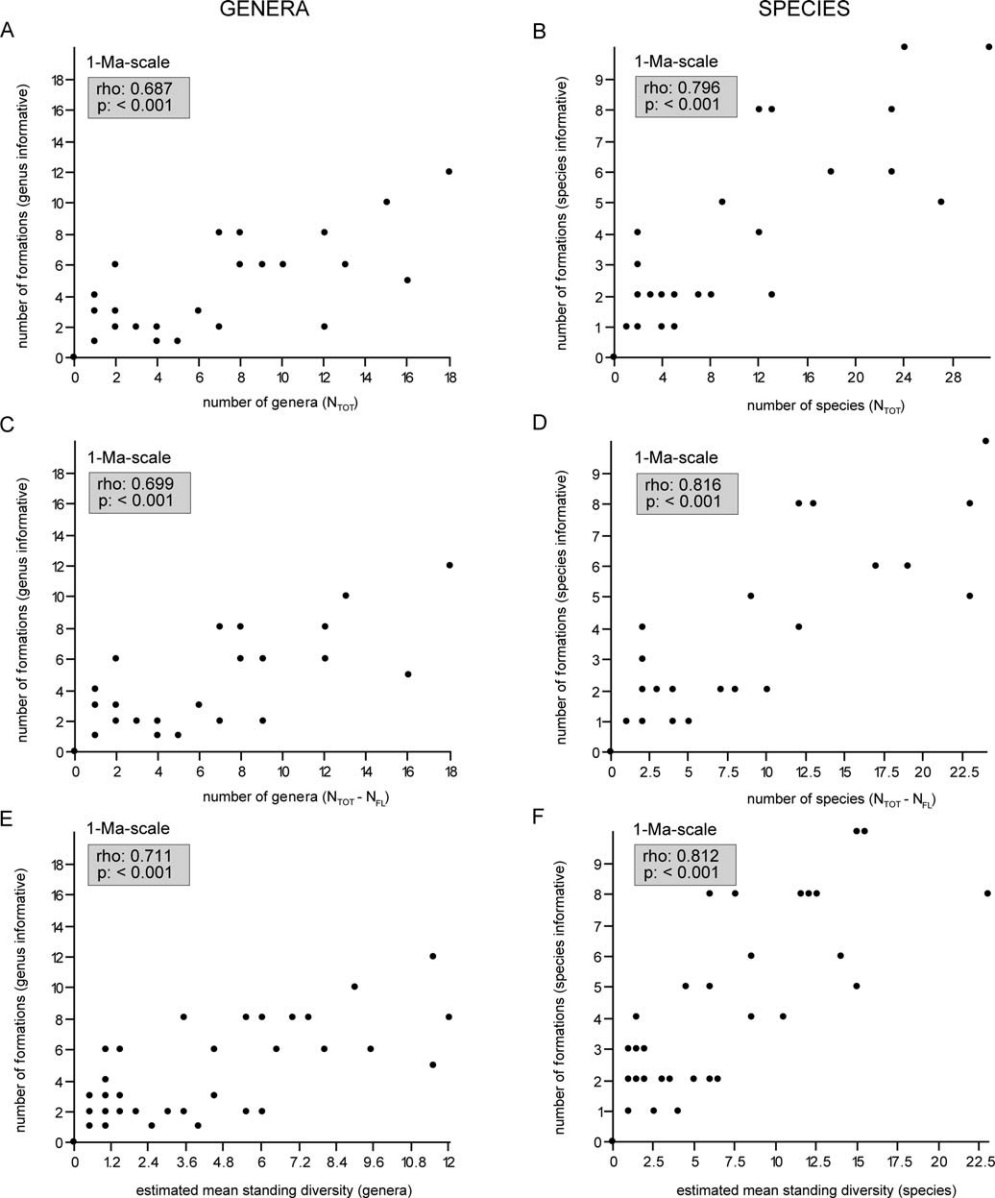
Correlation of anomodont diversity and the rock record at the 1-Ma-scale. Graphs plotting the number of genera (a, c, e) and species (b, d, f) against the number of informative formations. a–b) Total richness (N_tot_) at the 1-Ma-scale, c–d) total richness excluding singletons (N_tot_−N_FL_) at the 1-Ma-scale, and e–f) estimated mean standing diversity at the 1-Ma-scale. Spearman's rho and p-values of all variables indicate a statistically significant positive correlation between the number of taxa and the number of formations.

Consistent patterns are also reflected in the richness curves and associated number of formations on the genus level at the stage- and 1-Ma-scale as well as both genus and species level diversity curves at the LVF- and 5-Ma-scale (not illustrated). Moreover, a statistically significant positive correlation between the number of taxa and the number of formations per interval is apparent from scatter plots as well as Spearman's ρ values (Fig. 4, 5). The figures reflect a strong relation between the two variables, but further reveal some additional patterns that emerge from an examination of the obvious outliers of the plots. The most striking pattern is seen in the genus plots at the LVF-scale and the stage-scale (Fig. 4a, c). On the one hand, particularly obvious outliers with low richness despite a high number of known formations are from the earliest Triassic (Stage: Induan; LVF: Lootsbergian). On the other hand, obvious outliers with a high richness but only few known formations are from the Middle Permian (Stage: Capitanian; LVF: Gamkan I)(see below for discussion).

**Figure 6:**
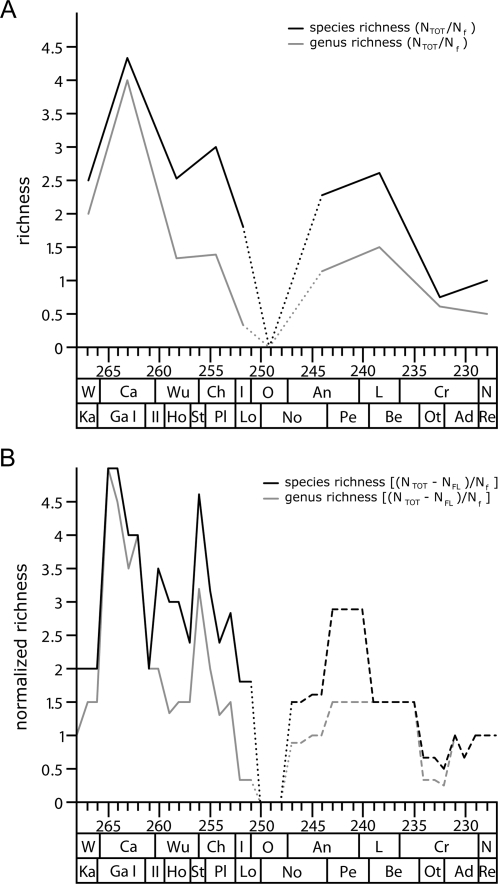
Normalized anomodont diversity. Genus and species diversity curve of a) total richness (N_TOT_/Δt) normalized by the number of informative formations (N_f_/Δt) at the stage-scale, and b) total richness excluding singletons (N_tot_−N_FL_) normalized by the number of informative formations (N_f_) at the 1-Ma-scale. Dotted lines indicate an artificial low in richness as a result of the gap in the fossil record of anomodonts in the late Early Triassic (Olenekian). Dashed lines indicate an artificially increased richness caused by the coarse correlation of Triassic anomodont faunas at the 1-Ma-scale.

To investigate to what extent the terrestrial rock record throughout the Permian and Triassic influences the observed diversity pattern of anomodonts, the total diversity and the total diversity without singletons was divided by the number of species-informative anomodont faunas at the stage-scale and at the1-Ma-scale, respectively (Fig. 6). These normalized diversity curves reflect a pattern quite different from that seen in the raw data, but it also contains familiar elements from the previous curves. The normalized pattern shows a rapid increase in diversity to an overall maximum already in the Capitanian (265.5 to 264.5 Ma). Thereafter, anomodont richness rapidly decreases to a genus low between 259.5 and 258.5 Ma and a species low slightly earlier (261.5 to 260.5 Ma). The genus curve then shows a continuous increase to the second highest peak between 256.5 and 255.5 Ma. In contrast, the species curve describes a more volatile pattern with another peak approximately at the level of the genus low, which is followed by a distinct decrease in diversity before an increase leads to the species maximum in the same interval as the genus peak. Subsequent to this maximum, anomodont richness declines rapidly to a genus low in the earliest Triassic (252.5 to 250.5 Ma), whereas the species low is not until the early Anisian (247.5 to 245.5 Ma) at the 1-Ma-scale. Thereafter, diversity increases to the Middle Triassic peak observed in the curves of the raw data. While the genus richness is stable throughout the entire Anisian, species richness is highest in the late Anisian, before the final decline of anomodonts.

In conclusion, the general pattern of the normalized curves implies a rapid initial diversification to the overall maximal genus and species richness at the end of the Middle Permian, followed by a continuous decline in diversity interrupted by the previously observed peaks in the latest Permian and during the Middle Triassic.

### Diversification and turnover in anomodont faunas

Taxonomic rates (origination and extinction) are important aspects in investigating the diversification and extinction of a clade, and respond quite differently to biases in the fossil record. Most importantly, Foote [79] demonstrated that the pattern of raw origination and extinction rates is real despite the possible influence of rock record biases, whereas the exact timing of peaks and lows are less reliable. The assumption that the pattern, albeit not the exact timing, of the raw data is real, is made here as well.

**Figure 7:**
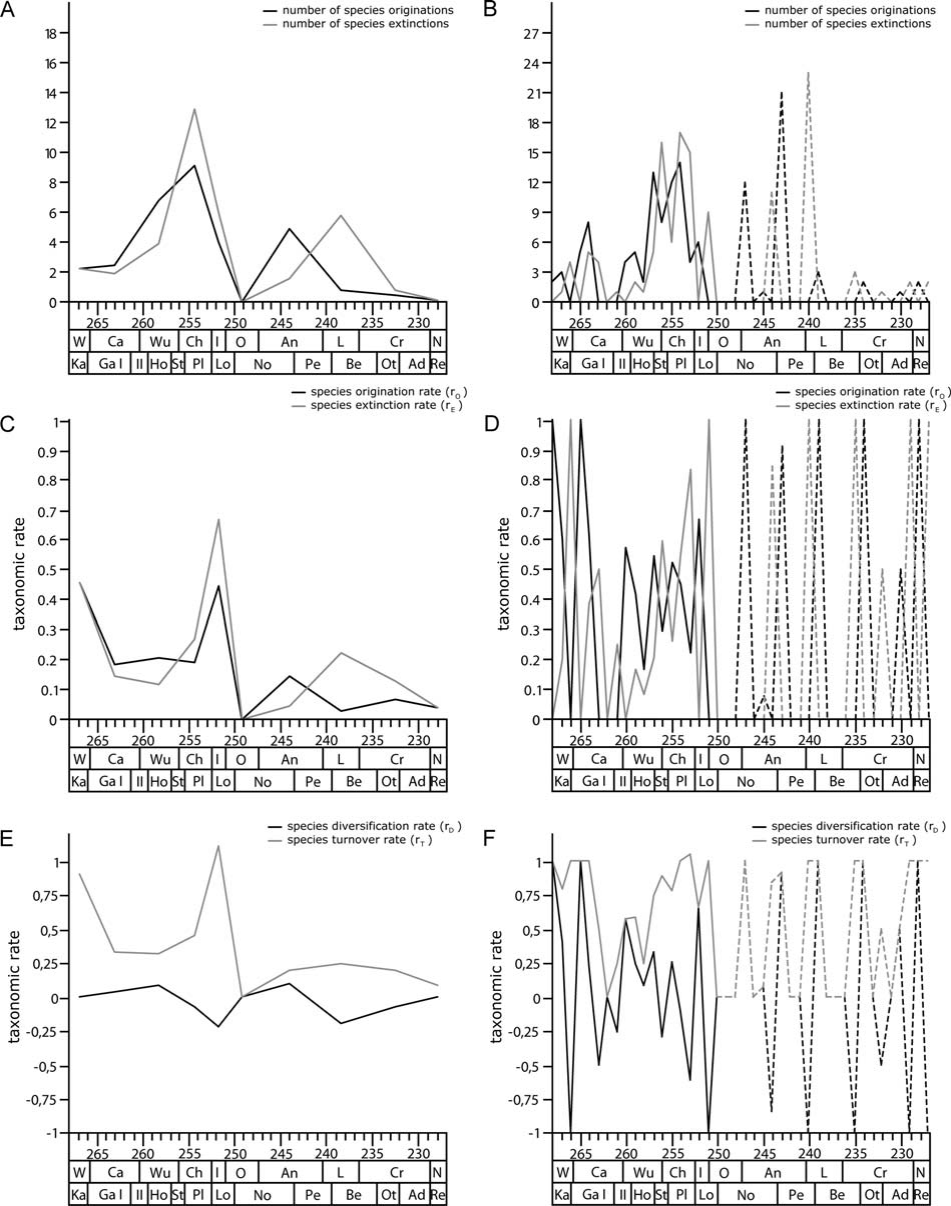
Taxonomic rates of anomodonts. Number of species originations (N_O_) and extinctions (N_E_), per-species origination (r_O_) and extinction rates (r_E_), and species diversification (r_D_) and turnover rates (r_T_) at the stage-scale (a, c, e) and at the 1-Ma-scale (b, d, f).

Figures 7a-b show the raw numbers of species originations and extinctions per stage and 1-Ma-interval. Genus originations and extinctions reflect a consistent pattern and are therefore not illustrated. The overall pattern of originations and extinctions shows a clear resemblance to the diversity curves illustrated in Figure 2a–f. An early increasing trend in the numbers of originations is followed by almost equal numbers of extinctions. At the 1-Ma-scale, there is a low in originations in the late Guadalupian (Capitanian), with a subsequent increasing trend towards the early Changhsingian and then again decreasing numbers in the latest Permian. The number of extinctions follows a similar trend, however, with the highest values in the latest Permian. Anomodonts are not known from the Olenekian, resulting in an artificially increased number of extinctions at its beginning and the origination of new species at the beginning of the Anisian. Thereafter, at the 1-Ma-scale, there are periodic peaks in origination and extinction at the end and beginning of each LVF, which are high throughout the Anisian but low in the late Middle and Late Triassic. Thus, there seems to be a correlation between the number of originations and the number of extinctions, however, not within one interval but rather with a slight delay in the peak of extinction following the origination. This delay is more pronounced in the Triassic than it is in the Permian and it is also seen at the stage-scale, which is likely the result of the less well-resolved stratigraphic ranges of the Triassic taxa.

The per-species origination and extinction rates as well as the resulting diversification and turnover rates are illustrated at the stage-scale and1-Ma-scale (Figs. 7c–f). The same pattern is also revealed by per-taxon rates on the genus level and the remaining time scales (not illustrated). At the 1-Ma-scale, Figure 7d shows an alternation between high origination and extinction rates in the Middle Permian, reflecting a high turnover rate in the early evolutionary history of anomodonts (see also Fig. 7e–f). The late Guadalupian (263.5 to 260.5 Ma) is characterized by a lack of originations, followed by fluctuating medium rates of origination during the Late Permian. In contrast, extinction rates decrease throughout the Middle Permian and thereafter continuously increase towards the latest Permian. Thereby, turnover rates are reduced in the early Late Permian but increase towards the Changhsingian. After the gap in the fossil record of anomodonts during the Olenekian, the Triassic is again characterized by an alternation of high origination and extinction rates, indicating an increased turnover rate during this time. The high degree in faunal turnover in the Middle Permian and during most of the Triassic is also reflected in the curve of diversification rates, which shows a consistent alternation between very high positive and negative values. In contrast, the diversification rates are strictly negative for an extended period of time after the end-Guadalupian extinction. Subsequently, the diversification rate switches to low but entirely positive values for the early Late Permian, which is followed by increasingly higher negative values towards the latest Permian and earliest Triassic, marking the end Permian extinction.

## Discussion

Diversity curves of the raw data indicate that anomodonts experienced three distinct diversifications with subsequent extinction events during their evolutionary history in the Permian and Triassic. The observed extinctions are at the end of the Guadalupian, at the end of the Permian, and close to the Anisian-Ladinian boundary. The former two of these extinction events have previously been documented on the basis of the tetrapod fossil record [7], [17], [18], [22], [26]. In addition, the mid-Permian event has recently been suggested to coincide with extinction of marine invertebrates at the end of the Guadalupian [49]. In contrast, the mid-Triassic event observed in this study seems to reflect an event unique to the fossil record of anomodonts (see below).

There is some variation in the observed diversity patterns based on the different diversity measures applied. Given the stratigraphic framework used as basis for this study, the diversity curve at the 1-Ma-scale shows an increased stratigraphic resolution and consequently a more accurate reflection of taxonomic richness, at least for the Permian. Moreover, except for the time-corrected richness curves at the LVF-scale and the stage-scale, the results of the diversity assessments on the different time scales are consistent in the recovery of the three events, respectively, suggesting a real signal at the end of the Guadalupian. Alternatively, the failure to recover the end-Guadalupian event at the time-corrected LVF-scale and the stage-scale might suggest that the detection of this event at the remaining scales potentially represents a relic of an apparent rather than a genuine increased stratigraphic resolution. Analyses on the genus and species level likewise reveal coherent patterns. The exclusion of singletons at the 1-Ma-scale results in lower richness peaks in the Middle and Late Permian but has no impact on the Triassic pattern. Diversity curves based on the estimated mean standing diversity (EMSD) recover the previously observed overall pattern of anomodont diversity but partially distort the relation between Permian and Triassic richness. This is the result of fundamental differences between the terrestrial rock records of these two periods. The overall maximum of anomodont diversity in the Triassic can be interpreted as a relic of the coarse correlation of global anomodont faunas, which are at the scale of the LVFs, whereas the Permian and earliest Triassic record is continuous and much better resolved as a result of the excellent sequence in South Africa. This includes the taxonomic uncertainty for Triassic anomodonts as well as the limited knowledge about the actual stratigraphic ranges of the Middle and Late Triassic anomodont taxa, causing an artificially high overall diversity during this time, which is indicated by the plateau in the Middle Triassic at the 1-Ma-scale (Fig. 2e). Thus, the turnover in the Triassic is concentrated at the boundaries between subsequent LVFs, even at the 1-Ma-scale, which results in artificially high originations and extinctions at those boundaries, a phenomenon that has previously been termed the Compiled Correlation Effect [80].

The impact of the end-Permian extinction on terrestrial tetrapod ecosystems has been hotly debated in the last decades. Early workers rejected the idea of an end-Permian extinction event on land simultaneous and similar in force to the marine realm, but rather argued for a gradual extinction of terrestrial tetrapods [6]–[8], [16], [18], [20]. Our current views of the dynamics of the end-Permian extinction in the terrestrial vertebrate ecosystem were largely shaped by the effort of two working groups [e.g. 2], [15], [22], [23]–[26], [81]–[84], who provided strong evidence for a sudden, possibly catastrophic event. Therefore, the gradual decline of anomodonts at the end of the Permian observed in this study most likely represents a result of the Signor-Lipps and Jaanusson effects, i.e. a smearing back of extinctions and smearing forward of originations, respectively [85]–[87].

The analysis of raw diversity data has often been challenged [e.g. 61], [74], [87], [88] as being biased by a number of factors. While some biases (see Material and Methods) are negligible, others (e.g. the rock record bias) have been shown to have a strong impact on diversity assessments of at least the marine realm [e.g. 71], [73], [74]. The present study investigated whether this concern also applies to the terrestrial tetrapod record across the PTB. Although previous studies on the local scales of the South African Karoo Basin and the Russian succession argued that rock record bias has no influence on the diversity pattern of terrestrial tetrapods [7], [24], the data presented in this study clearly demonstrate the influence of a rock record bias on diversity assessments of anomodonts on a global scale. This is based on a statistically significant positive correlation between the number of formations and the number of taxa in a given time interval (Figs. 3–5). Thus, there is an obvious rock record bias affecting the diversity curve of anomodonts during at least parts of the Permian and Triassic. However, an additional pattern emerges from the examination of obvious outliers in the scatter plots, in particular at the genus level of the stage-scale and LVF-scale (Figs. 4a, c). Obvious outliers with low richness despite high numbers of formations are of Early Triassic age, suggesting that the low diversity pattern following the Permian-Triassic extinction reflects a real signal notwithstanding the evident rock record bias. In contrast, obvious outliers with a high richness but only few known formations are seen in the Middle Permian. This potentially indicates the presence of a ‘Lagerstätten effect’, corresponding to a better sampling of biodiversity in one time bin relative to another (see below).

In an attempt to correct for this bias imposed by the variable rock record, the diversity curves of anomodonts were normalized by dividing the richness counts by the number of formations per time interval. These normalized diversity curves display previously observed elements but generally show a pattern that is quite different from that of the raw data. It reflects a rapid initial diversification to overall maxima of the genus and species richness at the end of the Middle Permian, being followed by a continuous decline in diversity only broken up by the peaks in the latest Permian and during the Middle Triassic (Fig. 6). Although a rapid initial diversification of anomodonts seems plausible in the light of the early radiation of basal therapsids in general [see 89], the pattern could alternatively represent a potential biogeographic phenomenon. The best anomodont fossil record of Middle Permian age is in South Africa, which coincides with their proposed centre of origin [e.g. 39]. As the greatest diversity of a clade can be expected close to the area of its origin, especially early in its history, the fossil record might have more densely sampled the early history of anomodont evolution as a matter of coincidence, resulting in an artificially increased early diversity of anomodonts.

It is important to note that diversity patterns and taxonomic rates (origination and extinction) respond differently to heterogeneity in the fossil record [63], [67]. For example, it has previously been recognized that although low diversity in the Early Triassic might be an artifact of a bias in the fossil record, this does not imply that there was not a significant turnover at the end of the Paleozoic [67], [72], [90], as it has been documented in ecological terms for terrestrial tetrapods at the PTB [24], [26]. Moreover, Foote [79] estimated true origination and extinction rates throughout the Phanerozoic based on observed first and last appearance dates and by considering variations in the incompleteness of the fossil record. His results suggest that most rate peaks in the raw data are real, whereas the exact timing of these peaks is less reliable [see also 69]. Therefore, it is here likewise assumed that the pattern, albeit not the exact timing, of the raw taxonomic rates is real.

The pattern of origination and extinction of anomodonts, as described above, reveals that the raw species originations and extinctions (Fig. 7a–b) closely resemble the raw diversity curves (Figs. 2a–f). Moreover, there seems to be a link between the number of originations and extinctions, although not within one time interval but rather with a minor delay of the extinction curve. As a result of the less well-resolved stratigraphic ranges of the Triassic taxa, this delay is more pronounced in the Triassic than it is in the Permian. Extended gaps between extinctions and a subsequent recovery (originations) in the fossil record of marine invertebrates throughout the Phanerozoic have been described as an artifact of the fossil record [69], rejecting earlier statements regarding a delayed recovery after background as well as mass extinctions [91]. With respect to the per-taxon origination and extinction rates as well as the resulting diversification and turnover rates (Figs. 7c–d, 7e–f), there is an alternation between high origination and extinction rates in the Middle Permian, describing a high turnover rate in the early evolutionary history of anomodonts. The end-Guadalupian extinction is the result of a low of originations in the late Guadalupian and continuing, albeit decreasing extinctions. The late Permian diversification is driven by medium to high origination rates and more importantly low extinction rates. In contrast, the end-Permian extinction is distinguished by unaltered medium to high origination rates, but even higher extinction rates. Consequently, turnover rates are relatively low in the early Late Permian and increase towards the Changhsingian. At the 1-Ma-scale, the Triassic shows an alternation of high origination and extinction rates, reflecting an increased turnover rate during this time. Thus, turnover rates are highest in the Middle Permian and during most of the Triassic, while decreased turnover rates in the Late Permian are possibly in part the result of higher species longevity of anomodonts during this time [92] (see also Fig. 1).

In conclusion, the pattern portrayed by the taxonomic rates of anomodonts indicates distinct changes in diversification and turnover dynamics throughout the Permian and Triassic. Anomodonts confirm the previously noted pattern that the end-Guadalupian and end-Permian extinctions were not simply driven by increased rates of extinction but also by low origination rates [26: 759,81,93]. However, this pattern is not evident at the final decline of anomodont diversity during the Middle Triassic. In contrast, taxonomic rates are equally unstable, throughout the entire period and the observed extinction in the Middle Triassic is not evident from the taxonomic rates alone. For this reason together with the apparent influence of a bias in the terrestrial rock record on the raw diversity curves, it remains unclear whether the Middle Triassic extinction represents an actual event or if it is a relic of our limited knowledge of the fossil record during this time. In any case, on the basis of the normalized diversity curves in combination with the pattern of taxonomic rates, it can be concluded that anomodonts experienced three distinct periods of diversification and at least two extinction events, i.e. the end-Guadalupian and end-Permian events, before their final decline towards the end of the Triassic. The end-Permian extinction represents the most distinct event in terms of decline in anomodont richness and turnover rates. Only further improvements to the stratigraphic framework and additional investigations on the diversity patterns of other Permian-Triassic terrestrial tetrapods will be able to reveal if the mid-Permian event corresponds to the end-Guadalupian event of the marine realm and whether the mid-Triassic decline of anomodont diversity represents a gradual or abrupt event that is unique to anomodonts or more common among terrestrial tetrapods. Ultimately, the incorporation of a phylogenetic perspective with the consideration of ghost-lineages at the species level will be an essential step to improve our understanding of the actual survivorship of anomodonts across the Permian-Triassic extinction event.

## Supporting Information

Text S1 Explanation of stratigraphic ranges of anomodont species from left to right as displayed in Figure 1.
(0.03 MB DOC)
