# Supplementary material for: Global Taxonomic Diversity of Anomodonts (Tetrapoda, Therapsida) and the Terrestrial Rock Record Across the Permian-Triassic Boundary
Source: PLoS One. 2008 Nov 17;3(11):e3733. doi: 10.1371/journal.pone.0003733 (PMC2581439; doi:10.1371/journal.pone.0003733)
Supplement: Text S1 — Explanation of stratigraphic ranges of anomodont species from left to right as displayed in Figure 1. (0.03 MB DOC) [file pone.0003733.s001.doc]

Text S1. Explanation of stratigraphic ranges of anomodont species from left to right as displayed in Figure 1.

*Eodicynodon oosthuizeni*

*‘Eodicynodon’ oelofseni*

*Patranomodon nyaphulii*

*Otsheria netzvetajevi*

*Venyukovia prima*

*Anomocephalus africanus*

*Colobodectes cluveri*

*Robertia broomiana*

*Pristerodon mackayi*

*Diictodon feliceps*

*Galechirus scholtzi*

*Brachyprosopus broomi*

*Lanthanostegus mohoii*

*Ulemica invisa*

*Ulemica efremovi*

*Galeops whaitsi*

*Chelydontops altidentalis*

*Emydops arctatus*

*Australobarbarus platycephalus*

*Australobarbarus kotelnitschi*

*Suminia getmanovi*

*Endothiodon uniseries*

*Endothiodon whaitsi*

*Endothiodon bathystoma*

*Pachytegos stockleyi*

*Tropidostoma microtrema*

*Emydops oweni*

*Cistecephalus microrhinus*

*Idelesaurus tataricus*

*Rhachiocephalus magnus*

*Oudenodon bainii*

*Galepus jouberti*

*Endothiodon mahalanobisi*

*Kingoria gracilis*

*Oudenodon grandis*

*Odontocyclops whaitsi*

*Aulacephalodon baini*

*Oudenodon sakamenensis*

*Kingoria howardi*

*Myosauroides minaari*

*Cistecephaloides boonstrai*

*Kitchinganomodon crassus*

*Kingoria recurvidens*

*Kingoria galecephala*

*Oudenodon luangwanensis*

*‘Dicynodon’ vanhoepeni*

*‘Dicynodon’ roberti*

*‘Dicynodon’ trigonocephalus*

*Dinanomodon rubidgei*

*Dicynodon lacerticeps*

*Kingoria grahami*

*Kingoria duvenhagei*

*Katumbia parringtoni*

*‘Dicynodon’ scopulusa*

*Elph borealis*

*‘Dicynodon’ amaltzkii*

*‘Dicynodon’ trautscholdi*

*Geikia locusticeps*

*‘Dicynodon’ huenei*

*‘Dicynodon’ bathyrhynchus*

*Pelanomodon rubidgei*

*‘Dicynodon’ leoniceps*

*Kingoria nowacki*

*Kawingasaurus fossilis*

*Rhachiocephalus behemoth*

*Propelanomodon devilliersi*

*Pelanomodon moschops*

*‘Dicynodon’ bogdaensis*

*‘Dicynodon’ limbus*

*Geikia elginensis*

*‘Dicynodon’ traquairi*

*‘Dicynodon’ sinkianensis*

*Interpresosaurus blomi*

*Delectosaurus arefjevi*

*Delectosaurus berezhanensis*

*Vivaxosaurus permirus*

*Kwazulusaurus shakai*

*Lystrosaurus maccaigi*

*Lystrosaurus curvatus*

*Lystrosaurus hedini*

*Lystrosaurus georgi*

*Lystrosaurus murrayi*

*Lystrosaurus shichanggouensis*

*Lystrosaurus robustus*

*Myosaurus gracilis*

*Lystrosaurus declivis*

*Kannemeyeria simocephalus*

*Kannemeyeria lophorhinus*

*Kannemeyeria xilougoensis*

*Kannemeyeria buerdongia*

*Kannemeyeria vjuschkovi*

*‘Kannemeyeria’ latirostris*

*Dolichuranus primaevus*

*Xiyukannemeyeria brevirostris*

*Rabidosaurus cristatus*

*Rhadiodromus klimovi*

*Vinceria argentinesis*

*Parakannemeyeria ningwuensis*

*Kombuisia frerensis*

*Vinceria andina*

*Rhinodicynodon gracile*

*Shansiodon wangi*

*Shansiodon wuhsiangensis*

*Shansiodon wupuensis*

*Shansiodon shaanbeiensis*

*Tetragonias njalilus*

# Wadiasaurus indicus

*Angonisaurus cruickshanki*

*Parakannemeyeria dolichocephala*

*Parakannemeyeria youngi*

*Parakannemeyeria shenmuensis*

*Parakannemeyeria chengi*

*Sinokannemeyeria pearsoni*

*Sinokannemeyeria yingchaoensis*

*Sinokannemeyeria sanchuanheensis*

*Rhadiodromus mariae*

*Rechnisaurus cristarhynchus*

*Sangusaurus edentatus*

*Sangusaurus parringtonii*

*Zambiasaurus submersus*

*Stahleckeria potens*

*Dinodontosaurus oliveirai*

*Jachaleria platygnathus*

*Placerias nmachouensis*

*Placerias hesternus*

*Ischigualastia jenseni*

*Jachaleria colorata*

*Jachaleria candelariensis*
